# A New Species of *Cleisostoma* (Orchidaceae) from the Hon Ba Nature Reserve in Vietnam: A Multidisciplinary Assessment

**DOI:** 10.1371/journal.pone.0150631

**Published:** 2016-03-23

**Authors:** Jan Ponert, Pavel Trávníček, Truong Ba Vuong, Romana Rybková, Jan Suda

**Affiliations:** 1 Prague Botanical Garden, Prague, Czech Republic; 2 Department of Experimental Plant Biology, Faculty of Science, Charles University in Prague, Prague, Czech Republic; 3 Department of Botany, Faculty of Science, Charles University in Prague, Prague, Czech Republic; 4 Institute of Botany, The Czech Academy of Sciences, Průhonice, Czech Republic; 5 Institute of Tropical Biology, Linh Trung Ward, Thu Duc District, Ho Chi Minh City, Vietnam; The National Orchid Conservation Center of China; The Orchid Conservation & Research Center of Shenzhen, CHINA

## Abstract

A new species, *Cleisostoma yersinii* J. Ponert & Vuong, is described and illustrated based on the material collected in the Hon Ba Nature Reserve in southern Vietnam. In addition to conventional (macro)morphological examination we comparatively investigated root and leaf anatomy (using light and fluorescent microscopy), assessed nectar characteristics (using HPLC analysis), determined nuclear genome size (using DNA flow cytometry) and reconstructed phylogenetic relationships (using nrITS sequences). *Cleisostoma yersinii* differs from its putative closest relative *C*. *birmanicum* in wider and shorter leaves, larger flowers, distinct lip with S-shaped tip of the mid-lobe, and a shallow spur with two large nectar sacks separated by prominent calli and septum. Nectar is sucrose-dominant and very rich in sugars. Stomata are developed on both sides of the leaf and have prominent hyperstomatal chambers and substomatal cavities. Roots with well-developed exodermis and tracheoidal idioblasts are covered by a two-layer *Vanda-*type velamen. Chloroplasts occur not only in the cortex but are also abundant in the stele. Mean 1C-value was estimated to 2.57 pg DNA. An updated identification key is provided for SE Asian sections and all Vietnamese species of *Cleisostoma*.

## Introduction

The genus *Cleisostoma* Blume is a taxonomically challenging group of orchids native to tropical and subtropical regions of the Indian Subcontinent, SE Asia, China and some of the Western Pacific islands [1]. The number of accepted species varies around one hundred [1,2], with the most recent estimate of 88 [3,4]. Molecular analyses support the placement of the genus into the subtribe Aeridinae, tribe Vandeae, subfamily Epidendroideae of Orchidaceae [3,5–9]. Despite its well-supported phylogenetic position, the traditional morphologically delimited genus *Cleisostoma* seems to be polyphyletic [3,5–7,9–10]. Aeridinae are one of the most complicated subtribes within Orchidaceae. The subtribe encompasses more than 1300 species in 83 genera [3], many of which are of notorious taxonomic difficulty [3,9,11,12]. Because robust phylogenetically-based taxonomic revision of the entire group is still lacking, we follow here conventional morphological circumscription of the genus, similarly to the most recent complete treatment of Orchidaceae [3].

Anatomical data may provide clues for phylogenetic relationships and taxonomic identifications because anatomy is usually less affected by environmental conditions than (macro)morphological characters. This promise has been fulfilled in several orchid groups (e.g., [13–17]), including the genus *Holcoglossum* from Aeridinae [18]. Possibly, anatomy may help to resolve taxonomic complexities in *Cleisostoma*, however, only little is known about anatomical variation in the tribe Vandeae.

Members of the tribe Vandeae usually have thick roots covered with a 1–8 cells wide velamen with well-developed pneumatodes [17,19,20]. Cells of the outermost velamen layer usually have much thinner walls than those of the endovelamen. This velamen structure seems to be unique for Vandeae and is referred to as the “*Vanda* type” [21]. Exodermis is always present, consisting of cells with thickened walls [17]. Tilosomes are usually lacking [17]; there is only one report of broadly lamellate tilosomes in *Saccolabium* sp. [22]. Endodermis is one-layered with O-thickened cell walls, exception being the thin-walled cells opposite xylem rays [17,20]. Leaves bear superficial stomata under hyperstomatal chambers (cuticular horns). Hypostomatic species seem to prevail over their amphistomatic counterparts in the Aeridinae [23]. Hypodermis is usually present (adaxial, abaxial or on both sides), but may be absent in some species [17,19].

**Fig 1:**
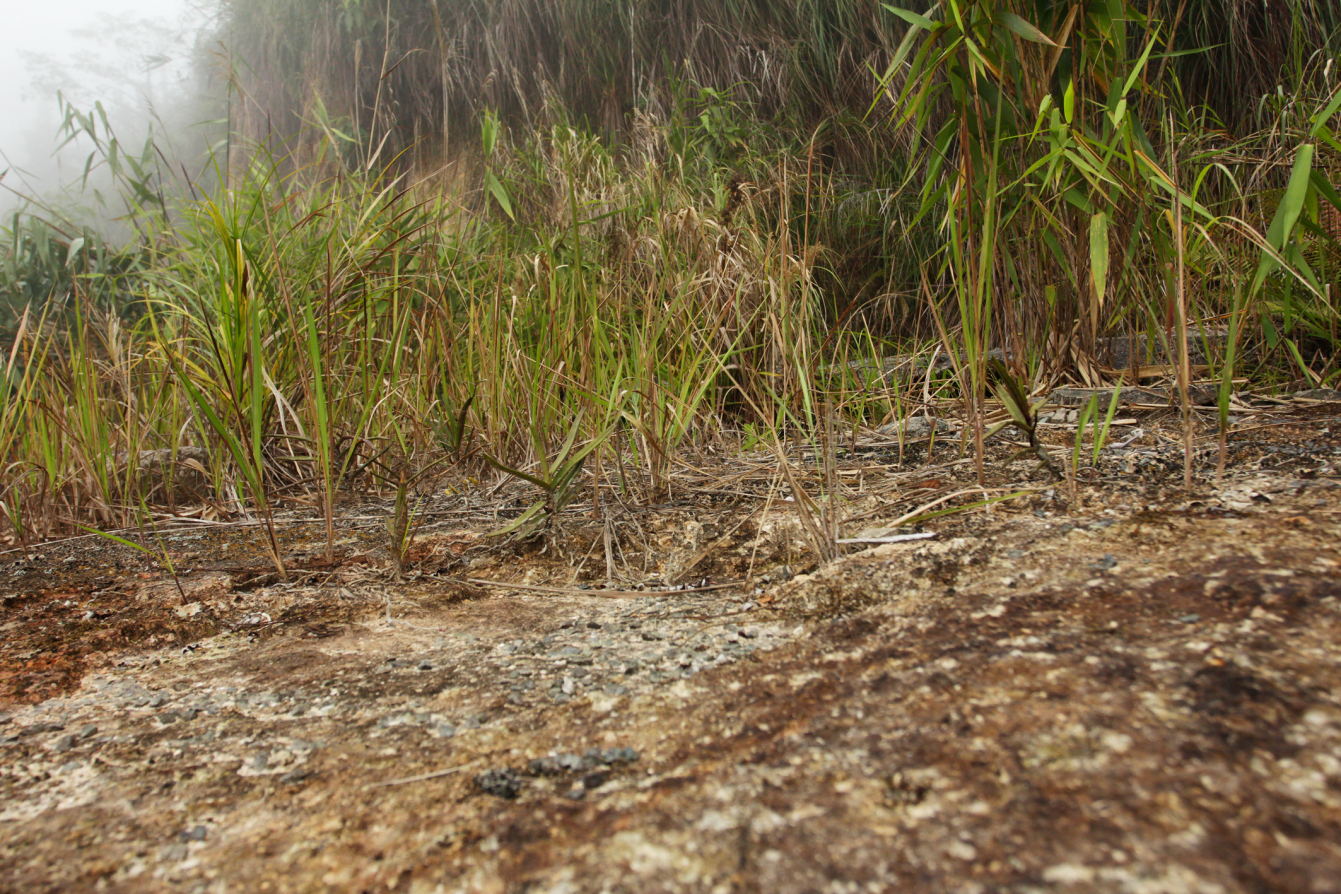
Habitat of *Cleisostoma yersinii*. Note numerous plants of *C*. *birmanicum* in the background. Photo J. Ponert.

**Fig 2:**
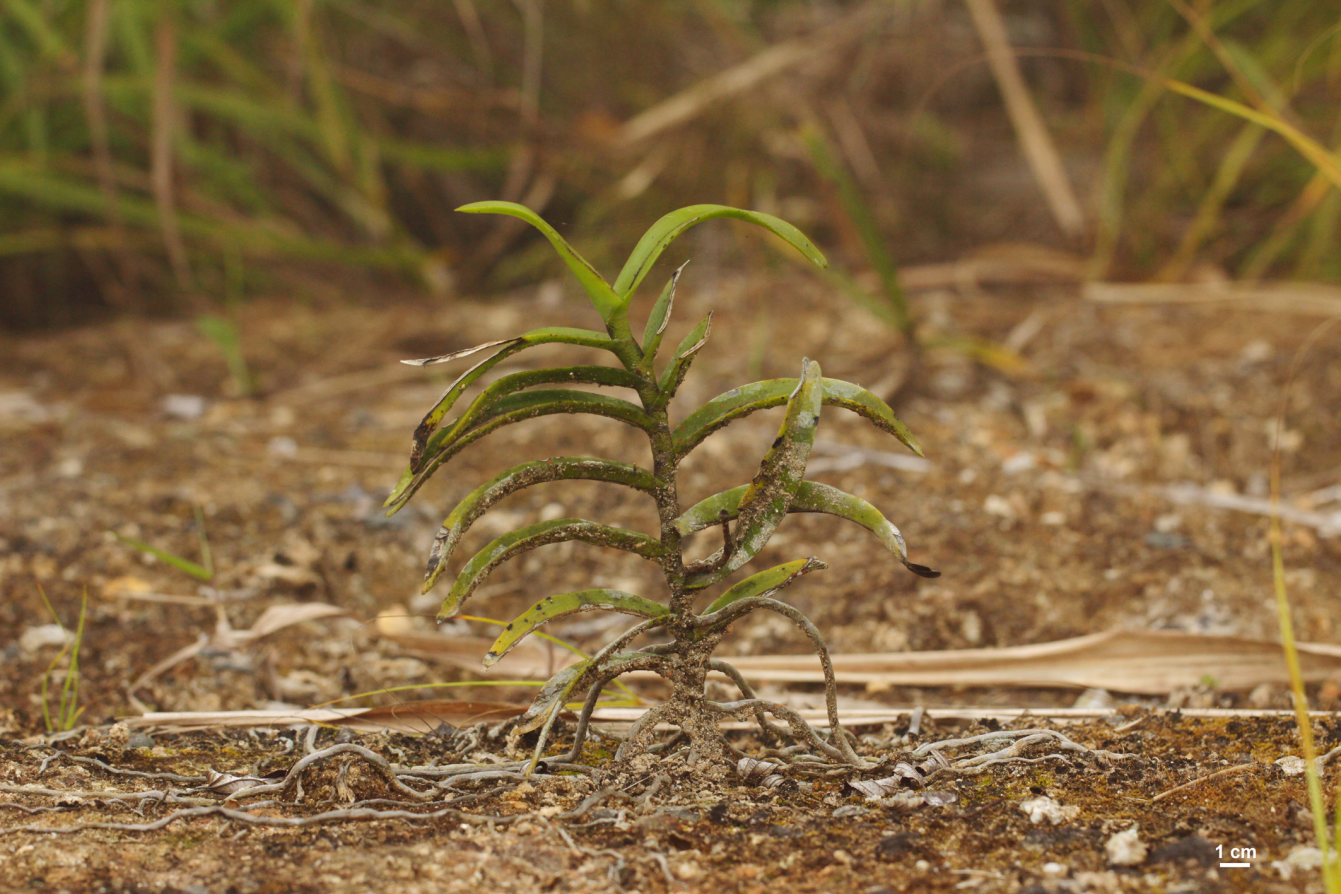
*Cleisostoma yersinii* growing on bare mineral soil. Photo J. Ponert.

Twenty-two *Cleisostoma* species have been reported from Vietnam [4,24–26]. However, considering the limited exploration of the Vietnamese flora, the total diversity may be higher and new species may await discovery [25]. One of the insufficiently explored areas is the Hon Ba Nature Reserve (19 165 ha) located in the Khánh Hòa Province, southern part of Vietnam, in the South Annamese floristic province [27]. The reserve mainly protects indigenous tropical forests and displays a high altitudinal range (spanning from near sea level up to 1 547 m a.s.l. in the Hon Ba peak). Although the summit of the Hon Ba peak can be easily reached by a road built in 2003, most parts of the reserve are hardly accessible due to steep topography. During a botanical survey of the reserve in March 2012, we found a rich orchid community along the road leading to the Hon Ba summit (Fig 1). Among others, we observed several non-flowering individuals of monopodial species that could not be identified on the spot and one individual was therefore taken into culture (Fig 2). When the plant flowered it was immediately obvious that it represents a new species. Morphological evidence (four pollinia in two masses in particular) suggested that the species belongs to genus *Cleisostoma*. It is formally described here as *C*. *yersinii* and its anatomy, morphology, nrITS sequence and genome size compared with other related species.

## Materials and Methods

### Ethics statement

Plants reported in this work were collected in the Hon Ba Nature Reserve in Vietnam in cooperation of the Institute of Tropical Biology (Vietnam), the Hon Ba Nature Reserve management (Vietnam) and the Prague Botanical Garden (Czech Republic) as previously agreed in the Memorandum of Cooperation. Permissions to enter the Hon Ba Nature Reserve and to collect the samples were issued by respective Vietnamese authorities. Transport of plant material from Vietnam to the Czech Republic was permitted by respective CITES authorities (CITES permission No: 12CZ022452).

### Cultivation conditions

Plant was collected in the Hon Ba Nature Reserve in Vietnam in 2012 and stored in a paper bag until planting in the Prague Botanical Garden, Czech Republic. It was grown in a greenhouse in plastic pots filled with pieces (2–3 cm large) of stone pine (*Pinus pinea* L.) bark. Pot was kept in full sun and high air humidity, with day / night temperatures 20–25 / 16–20°C. Pot was allowed to dry out between watering. Pictures of a flowering plant were taken using a digital camera with a macro lens (Canon EOS 60D equipped with Canon Macro EF 100 mm 1:2,8 L IS USM and extension tubes for greater magnification) mounted on a tripod (to achieve the same focus distance and identical scale for all pictures) or using the same digital camera mounted on a stereomicroscope Olympus SZ X7 as detailed in [28].

### HPLC analysis of floral nectar

Nectar sacks were dissected by a razor blade and the nectar was allowed to flow out of the lip into a petri dish, immediately weighted, diluted by 300 μl of Milli-Q ultrapure water (Millipore, Bedford, MA, USA) and filtered through a 0.45 μm membrane filter (Millipore) into a microtube. The samples were kept in a freezer at -20°C until the HPLC analysis. Nectar sugar composition was determined by an HPLC system with refractometric detection (Spectra Physics; refractometer Shodex RI-71; integrator ChromJet; pre-column filled with IEX Pb form 8 μm, column 250 x 8 mm filled with IEX Pb form 8 μm; Watrex, Prague, Czech Republic) following the protocol of [29]. One nectar sample from each of the three flowers was separately analyzed. To calculate the total volume of nectar, aqueous solution of sucrose, glucose and fructose of the same concentration as detected in nectar was prepared and its density was measured.

### Anatomical study

Root and leaf tissue was sectioned into thin slices using a hand microtome and observed either in transmitted light under a microscope Olympus BX50 equipped with Nikon DS-5M camera or in autofluorescence spectra or Nomarski differential contrast under an epifluorescence microscope Olympus Provis AX70 equipped with Nikon DS-Fi1 camera. Excitation filters BP 330–385 and BP 510–550 were used for UV-induced autofluorescence and green light-induced autofluorescence, respectively.

### Stomatal density

Surfaces of three fully developed leaves per species were observed in UV-induced fluorescence mode under the microscope Olympus Provis AX70 as described above. Each leaf was divided into 1 cm long segments (from the tip to the base), the basal and apical ones were discarded, and stomatal densities in the remaining segments were estimated using a randomly placed 1 mm^2^ sampling window [30,31]. For comparative purposes, specimens of *C*. *birmanicum* (Schltr.) Garay, *C*. *paniculatum* (Ker Gawl.) Garay and *C*. *racemiferum* (Lindl.) Garay cultivated in the Prague Botanical Garden were included.

Interspecific differences in stomatal densities and differences between adaxial and abaxial sides of the leaf were tested by ANOVA, followed by the Tukey-Kramer test [32] using the R 2.9.1. package [33]. Adaxial sides of species lacking any stomata were excluded from statistical comparisons.

### Genome size estimation

Holoploid genome sizes (C-values) were estimated using DNA flow cytometry following the simplified two-step protocol using Otto buffers as detailed in [34]. Intact nuclei were isolated from apical parts of young leaves, stained with intercalating fluorochrome propidium iodide and the fluorescence intensity of 5000 particles was recorded on a Partec CyFlow SL (Partec-Sysmex, Münster, Germany) cytometer equipped with a 532 nm, 150 W output power Cobolt Samba laser (Cobolt, Solna, Sweden). Most samples were re-analyzed on different days to control for potential random shifts in instrument measurements. *Pisum sativum* 'Ctirad' (1C = 4.38 pg) [35] was used as an internal reference standard.

### Molecular phylogeny

Leaf samples of three *Cleisostoma* species (see S1 Table) were collected from living plants cultivated in the Prague Botanical Garden and immediately dried in silica gel. Total genomic DNA was extracted using the DNeasy 96 Plant kit (Qiagen, Venlo, The Netherlands) following the manufacturer´s instructions. Polymerase chain reaction (PCR) amplifications were carried out as described in [7] and [9] using the set of oligonucleotide primers used by [9]. Similarity searches against the GenBank database were performed with the obtained sequence data using the Blast+ algorithm [36] in order to check for potential fungal contamination and find sequences of related species. All sequences of the “*Cleisostoma* clade” from a recent molecular phylogeny of Aeridinae [9] were downloaded from GenBank and included in phylogenetic analysis. In total, 230 sequences of 56 species were used.

Sequences were aligned in ClustalW2 [37] and manually edited in BioEdit 7.2.5. Phylogenetic analyses based on maximum parsimony (MP), Bayesian inference (BI) and Maximum likelihood (ML) were performed with PAUP⁄ version 4.0b10 [38], MrBayes 3.2.2 [39], and Garli 2.01 [40], respectively. Missing data were coded with ‘‘?” and the gaps were coded in SeqState 1.4.1 [41] according to approach of Simmons and Ochoterena [42]. In the MP analyses, all of the characters were equally weighed and unordered, and a heuristic search with 1000 random addition sequence replicates and tree-bi-section-reconnection branch swapping were performed. For the BI and ML analyses, the best-fit models for each partition were selected by jModelTest2.1.7 [43] under the Akaike Information Criterion. BI was performed under the following settings: sampling frequency = 100, temp = 0.1, burn-in = 10,000, and number of Markov Chain Monte Carlo generations = 1,000,000. For ML analysis he “genthreshfortopoterm” option was set to 100,000 and branch support was assessed with 200 bootstrap replicates under the same criteria. All new sequences were submitted to GenBank (see S1 Table).

### Nomenclature

The electronic version of this article in Portable Document Format (PDF) in a work with an ISSN or ISBN will represent a published work according to the International Code of Nomenclature for algae, fungi, and plants, and hence the new names contained in the electronic publication of a PLOS ONE article are effectively published under that Code from the electronic edition alone, so there is no longer any need to provide printed copies.

In addition, new names contained in this work have been submitted to IPNI, from where they will be made available to the Global Names Index. The IPNI LSIDs can be resolved and the associated information viewed through any standard web browser by appending the LSID contained in this publication to the prefix http://ipni.org/. The online version of this work is archived and available from the following digital repositories: PubMed Central, LOCKSS.

## Results

### Taxonomic treatment

**Fig 3:**
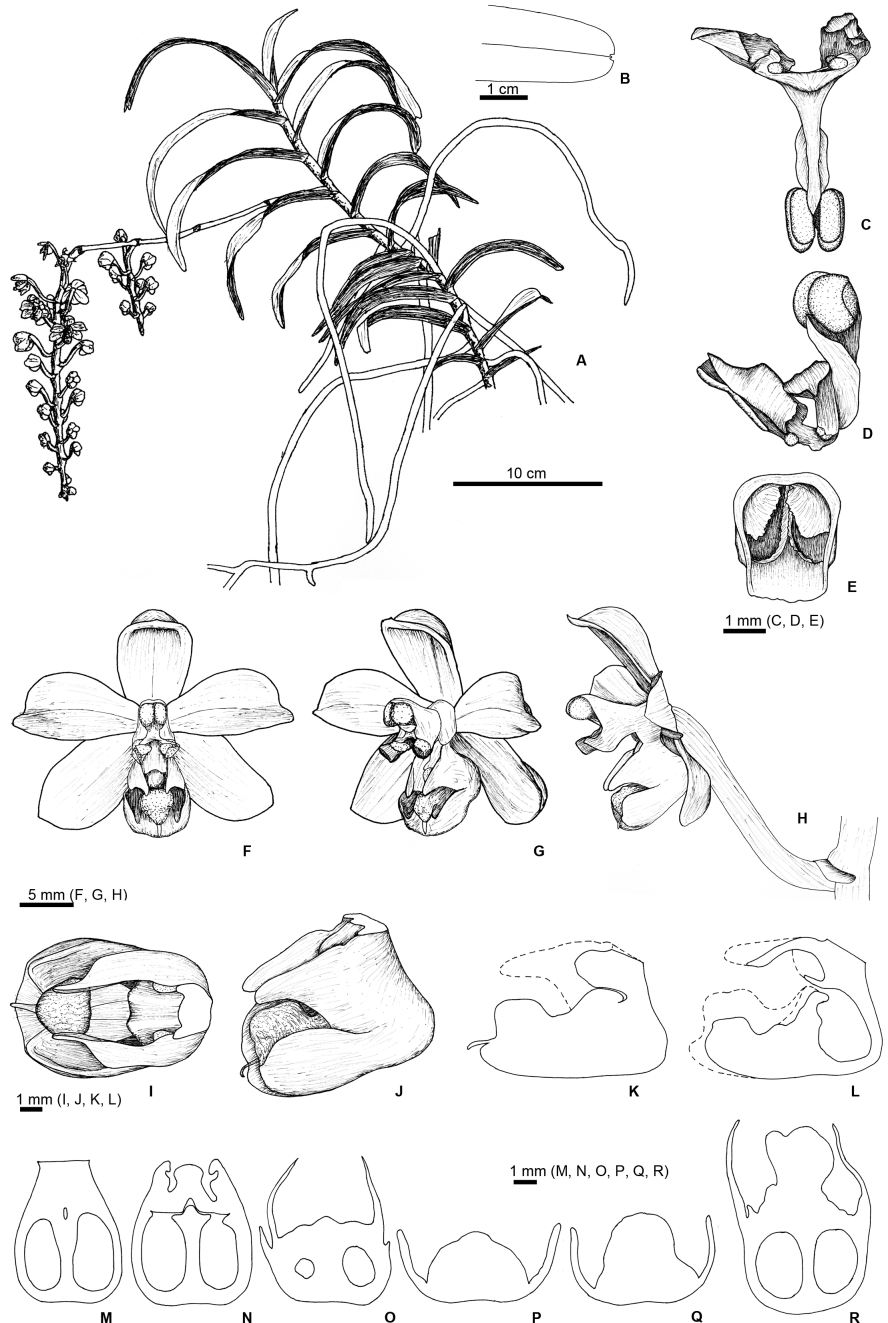
Drawings of *Cleisostoma yersinii*. (A) Habit. (B) Apical part of the leaf. (C) Pollinarium (front view) removed from the flower. (D) Pollinarium (side view). (E) Cap. (F–H) Flower from different angles. (I) Lip from above. (J) Lip from the side. (K) Vertical longitudinal section through the middle of the lip. (L) Vertical longitudinal section of the lip in one quarter of the lip width. (M.-Q) Vertical transverse sections of the lip, from the base to the tip. (R) Horizontal longitudinal section through the middle of the lip (parallel to the bottom side). Drawn by J. Ponert after the holotype.

**Fig 4:**
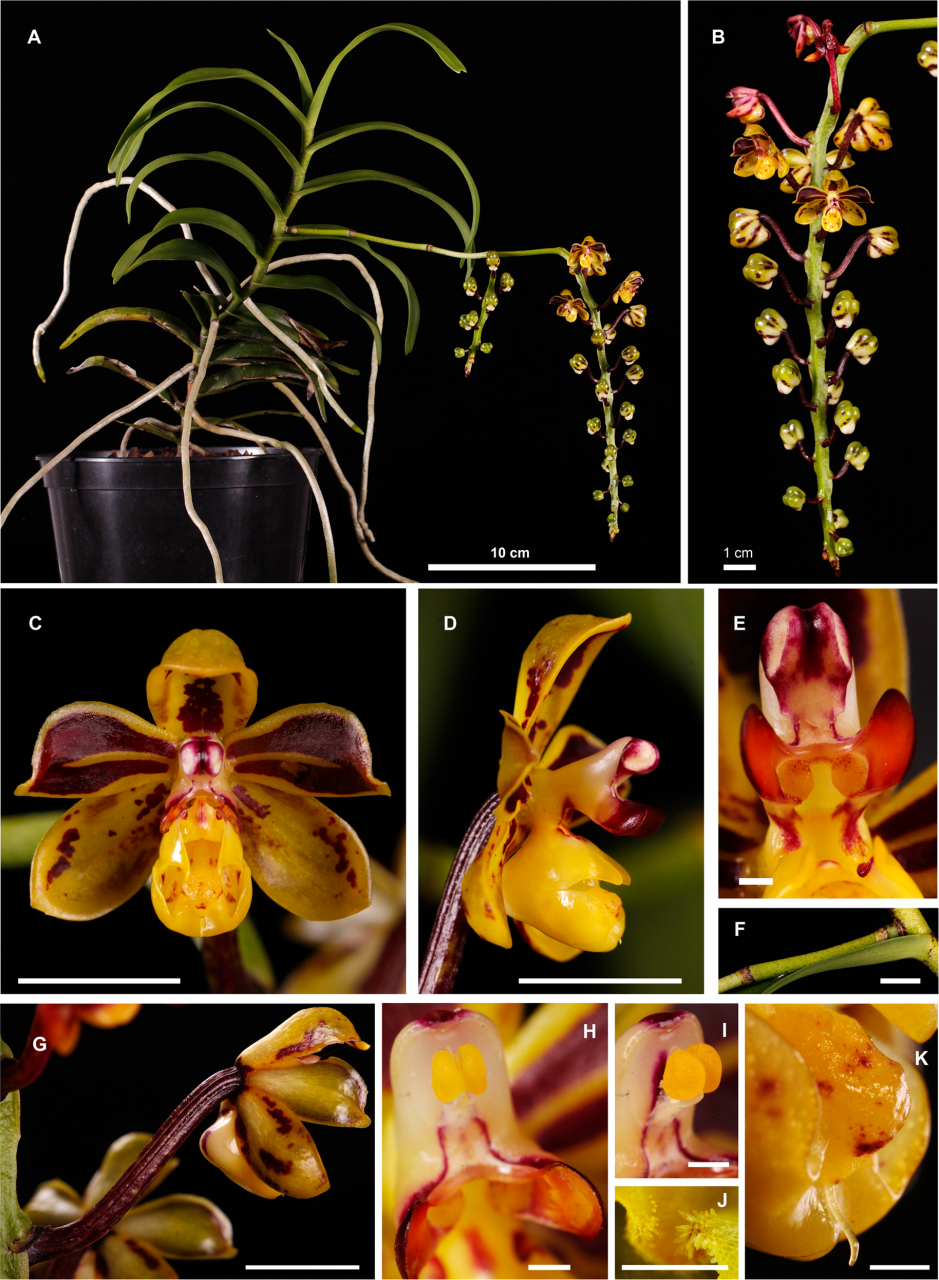
Pictures of *Cleisostoma yersinii*. (A) Habit. (B) Inflorescence. (C) Flower from the front. (D) Flower from the side. (E) Column from the front. (F) Peduncle. (G) Ovary. (H) Column with a removed cap. (I) Pollinarium on the column with a removed cap. (J) Papillate surfaces of lip calluses. (K) Tip of the lip with a single tail (arista). Scale bars: C, D, F, G– 1 cm; E, H, I, J, K– 1 mm. A specimen cultivated in the Prague Botanical Garden collected as holotype. Photo J. Ponert.

***Cleisostoma yersinii*** J. Ponert & Vuong, *sp*. *nov*. [urn:lsid:ipni.org: names: 77153287–1] (Figs 3 and 4)

Species resembling *C*. *birmanicum* but differing in wider and shorter leaves, larger flowers, lip with large central and backwall calli, short and wide spur, and two nectar sacks separated by prominent calli and a well-developed septum. Mid-lobe of the lip divided into three lobules, the lateral ones bent upwards, the central one S-shaped, projecting into a single tail.

**Holotype:—**A specimen cultivated in the Prague Botanical Garden, the Czech Republic, pressed on 8 September 2013, *Jan Ponert 674* (PRC!: 455377). Originally collected in Vietnam, Khánh Hòa province, Cam Lâm District, Hòn Bà Nature Reserve, roadside in submontane cloud forest, alt. 1380 m, 7. 3. 2012, leg. *J*. *Ponert*, *T*. *Q*. *Tam*, *T*. *B*. *Vuong*, *R*. *Rybková*, *P*. *Vacík & K*. *Petrželka*.

**Syntype:**—A cultivated specimen from the same collection, pressed on 24 September 2014, *Jan Ponert 717* (PRC!: 455378)

### Description

**Fig 5:**
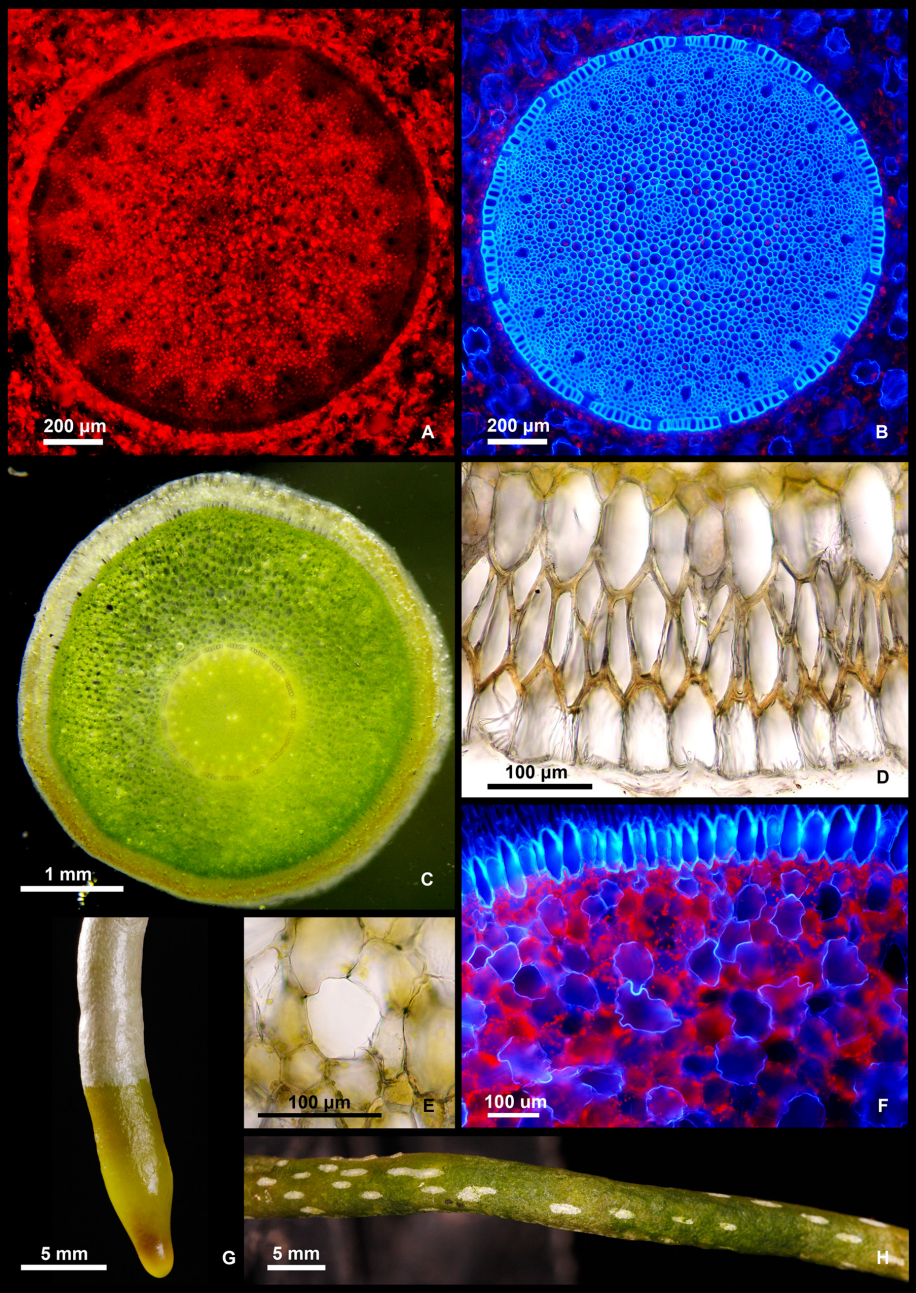
Roots of *Cleisostoma yersinii*. (A) Cross-section of the stele, green-excited autofluorescence showing the distribution of chloroplasts (in red). (B) The same section as in A, UV-excited autofluorescence showing anatomical details, including phloem and xylem strands and endodermis with passage cells. (C) Cross-section of the root. (D) Two-layered velamen with exodermis. (E) Tracheoidal idioblast (water storage cell) in root cortex. (F) Root cortex with exodermis (blue) and numerous tracheoidal idioblasts (UV-excited autofluorescence). (G) Growing tip of a root. (H). Wet root showing white pneumatodes in translucent (green) velamen.

Perennial with monopodial growth. Stem 0.7–0.8 cm in diameter, up to 30 cm in studied specimens but most likely longer in mature plants in the field, with 1–2 cm long internodes, entirely clothed by tubular leaf sheaths. Roots adventitious, aerial, glabrous, 4.0–5.0 mm in diameter, covered by two-layered velamen. Velamen white in dry state but translucent when wet, revealing the green cortex; pneumatodes linear-elliptic, white (Fig 5H). Growing tips of roots green, occasionally with violet tinge in the meristematic part (Fig 5G). Leaves alternate, distichous. Leaf blades thick, conduplicate, glabrous, narrowly oblong, 85–155 × 18–21 mm, with emarginate and unequally bilobed apex. Leaf sheaths glabrous, bullate, green, with dark reddish-brown spots on the surface opposite to the leaf blade. Inflorescence racemose, branched, overtopping leaves, glabrous. Peduncles green, 3.0–3.5 mm in diameter, up to 135 mm long, with four, 2.0–4.0 mm long, green, reddish-brown marked bracts. Rachis pendulous, green, 2.5–3.0 mm in diameter, 100–170 mm long, bearing 8–22 flowers. Flowers large, widely opening, up to 27 mm wide, subtended by small, 1.0–1.8 mm long, dark-colored bracts, adnate to the ovary. Ovary resupinated, dark reddish-brown, 20–25 mm long. Lateral sepals obtuse-obovate, up to 14 × 7 mm, slightly concave, yellow with reddish-brown dots. Dorsal sepal obtuse-obovate, up to 13 × 6 mm, concave, yellow with reddish-brown markings. Petals obtuse, up to 13 × 6.5 mm, reddish-brown, with a narrow (less than 1.0 mm) yellow margin and a yellow stripe along the midvein, not reaching the yellow margin. Lip yellow, up to 9 × 6.5 mm, 3-lobed, with a large central callus (protuberance), a smaller callus at the base of the lip (backwall callus), and a well-developed longitudinal septum connected with calli and completely dividing the very shallow spur into two separate sacks filled with nectar. Lateral lip lobes triangular, upward-pointing, slightly oblique in upper parts. Mid-lobe of the lip shallowly divided into 3 lobules; the lateral ones very short and wide, pointing upwards; the central lobule S-shaped, curved backwards, forming a large callus at the tip, with a narrow triangular projection extending to a single arista (tail) (Figs 3I, 3J and 3K and S1 Fig). Callus in the central lobule connected with the large central callus of the lip by a narrow neck (Fig 3K and 3L). Central callus tightly attached to the small backwall callus, leaving two narrow openings by which nectar sacs can be accessed by pollinators. Surfaces of the calli around the openings papillate. All visible parts of the lip glabrous and shiny, except for upper parts of apical and central calli that are bullate. Column up to 8 mm long, at the base with a large flat rostellum projection (rostellar wings or rostellar arms) on either side. Pollinarium single, with stipes deeply divided at the tip with viscidium (hippocrepiforme), four yellow pollinia arranged in two separated masses. Cap shiny, dark purplish red, with two large white circles above the pollinia.

### Etymology

The species is named after Alexandre Emile Jean Yersin (1863–1943), a Swiss-French physician and bacteriologist who significantly contributed to the exploration of the Hon Ba mountain area.

### Ecology

Plants were found growing on a roadside at elevation about 1380 m a.s.l. The surrounding vegetation was formed by a primary submontane evergreen forest, with many epiphytes. During our field work (February–March) the site was humid with high soil and air moisture, and frequent clouds. Plants were growing directly on bare mineral soil (clayey-sandy weathered residue of granite) exposed during the building of the road in 2003. *Cleisostoma* roots spread along the ground surface, occasionally reaching more than 1 m. Surface of the soil was largely bare, with only a few lichens and mosses.

*Ophioglossum reticulatum* L. and *Psilotum nudum* (L.) P.Beauv. were the most abundant species recorded on the site. Other orchids growing in sympatry were *Pholidota leveilleana* Schltr. (*R*. *Rybková*, *T*. *Q*. *Tam*, *T*. *B*. *Vuong*, *J*. *Ponert HB-212*, 7.3.2012, PRN, VNM), *Cleisostoma birmanicum* (*J*. *Leong-Škorničková*, *R*. *Rybková*, *J*. *Ponert*, *H*. *Đ*. *Trần HB-74*, 3.7.2011, PRN, VNM, SING), *Anoectochilus annamensis* Aver., *Cymbidium erythrostylum* Rolfe, *Dendrobium uniflorum* Griff., *Thrixspermum annamense* (Guillaumin) Garay and *Thrixspermum* sp. (cf. *T*. *centipeda* Lour., flowers not seen).

Another individual of *Cleisostoma yersinii* in full flower was found by TBV at elevation about 1500 m a.s.l. in September 2014 (S2 Fig). Plant was growing as an epiphyte on tree trunk in a primary submontane evergreen forest.

### Breeding system

Fruit developed only from hand-pollinated flower, suggesting that the species is allogamous.

### Nectar HPLC analysis

Nectar was very rich in total sugar content, reaching 0.58 ± 0.09 mg (mean ± SD) of saccharides per mg of nectar, which corresponds to 74.51 ± 11.48% (w/v). Three different saccharides were detected: sucrose, glucose and fructose (S3 Fig). Sucrose was clearly dominant in the spectrum (95.11 ± 0.83%, mean ± SD) while glucose and fructose were present as minorities at proportions 2.82 ± 0.55% and 2.07 ± 0.28%, respectively.

### Root anatomy

The velamen is two cells wide, of the *Vanda* type (Fig 5D); the lower layer (endovelamen) with O-thickened cell walls, the outer layer (epivelamen) with thin cell walls. Cells of epivelamen may elongate and form root hairs when in contact with bark surface. Pneumatodes in velamen linear-elliptic, 1–7 mm long, arranged in longitudinal lines (Fig 5H).

Exodermis cells are radially elongated with ∩-thickened walls, impregnated by lignin-like compounds, especially in thickened cell walls adjacent to velamen (Fig 5D and 5F). Cortex chlorenchymatous, containing numerous large non-living tracheoidal idioblasts (also referred to as water-storage cells) with lignified cell walls (Fig 5E and 5F). Endodermis one-layered, cells isodiametric to slightly radially elongated, cell walls O-thickened and lignified except of the passage cells opposite to xylem rays (Fig 5B).

Vascular cylinder 17-arch in studied roots, with alternating xylem and phloem strands (Fig 5A and 5B) embedded in parenchymatous tissue, sclerenchymatous around the phloem. Pith with numerous chloroplasts, which are almost absent in sclerenchymatous tissue around phloem strands (Fig 5A and 5C).

### Leaf anatomy

**Fig 6:**
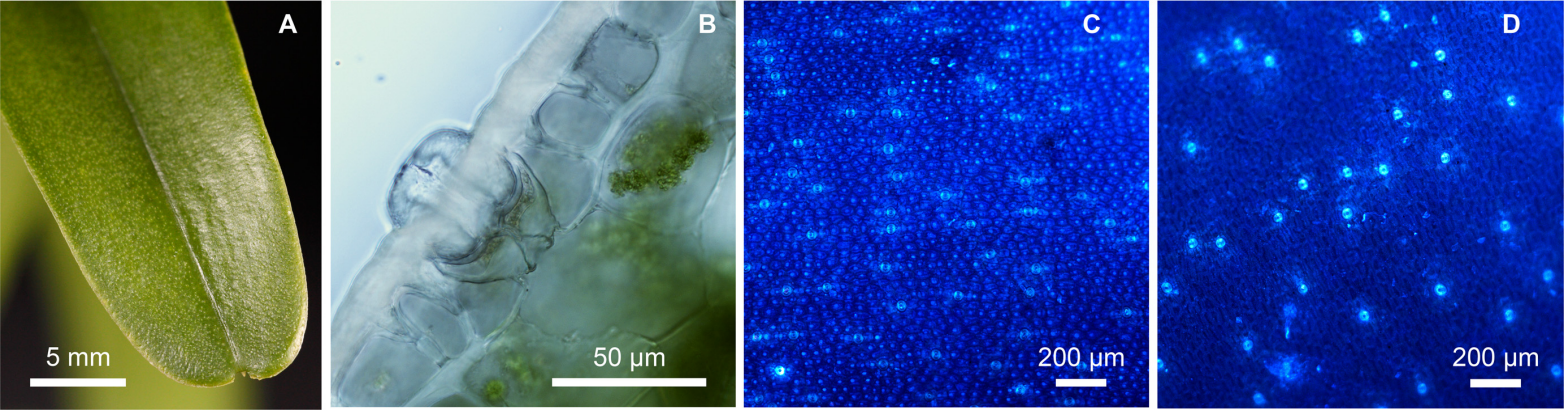
Leaves of *Cleisostoma yersinii*. (A) Apical part of the leaf. (B) Hyperstomatal chamber with stoma and substomatal cavity below (adaxial epidermis, Nomarski difference contrast). (C) Abaxial epidermis with stomata (UV-excited autofluorescence). (D) Adaxial epidermis with stomata (UV-excited autofluorescence).

Cuticle smooth on the adaxial side, ridged on the abaxial side, 11–18 μm thick. Epidermal cells periclinal. Stomata superficial, guard cells of each stoma are positioned below a relatively large hyperstomatal chamber and directly associated with a large substomatal cavity (Fig 6B). Hypodermis one-layered, present on both sides of the leaf, resembling water-storage cells. Mesophyll heterogeneous with water-storage idioblasts. Vascular bundles collateral, in one row.

**Fig 7:**
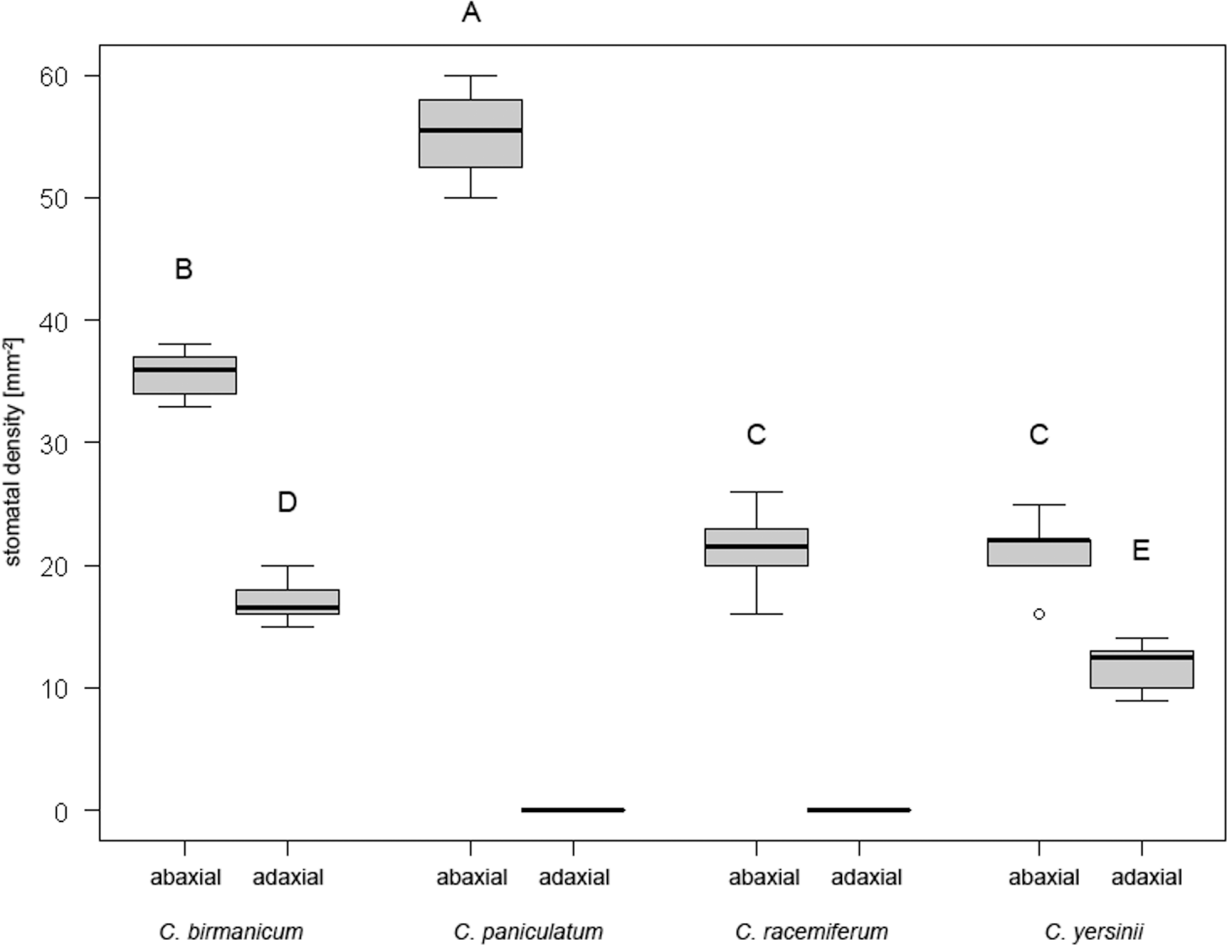
Box-and-whisker plots showing leaf stomatal densities of different *Cleisostoma* species. Different letters indicate significantly different groups according to ANOVA followed by the Tukey-Kramer test.

Stomata present on both sides of leaves, more abundant on the abaxial (mean 21.3 mm^-2^; Fig 6C) than on the adaxial side (mean 12.0 mm^-2^; Fig 6D). The same pattern in stomata distribution was observed in *Cleisostoma birmanicum*, while leaves of *C*. *racemiferum* and *C*. *paniculatum* bear stomata on the abaxial side only (Fig 7). Stomatal density differs significantly among the studied groups (one-way ANOVA: F_(5,49)_ = 370.98, p < 0.001, η^2^ = 0.97). The highest density on the abaxial side was observed in *C*. *paniculatum* while *C*. *racemiferum* and *C*. *yersinii* had the lowest density (Fig 7).

### Nuclear genome size

Flow-cytometric analyses resulted in high-resolution fluorescence histograms with distinct peaks of both the studied sample and internal reference standard (coefficients of variation of G0/G1 fluorescence peaks below 4%) and little background. 1C-values of seven analyzed species ranged from 1.80 pg to 2.67 pg, spanning nearly 1.5-fold range (Table 1). There were three significantly different (ANOVA, F = 205.5, p < 0.001) groups of holoploid genome sizes: 1) *C*. *racemiferum*, 2) *S*. *subulatum* Blume, and 3) the remaining five species.

**Table 1:** 1C-values of selected *Cleisostoma* species as estimated using propidium iodide flow cytometry.

Species	2C-value ± SD [pg DNA]	1C-value [pg DNA]	1C-value [Mbp]	N*	Tukey HSD group
***C*. *racemiferum***	3.60 ± 0.08	1.80	1760.4	6	A
***C*. *subulatum***	4.51 ± 0.08	2.25	2200.5	4	B
***C*. *paniculatum***	4.97 ± 0.11	2.49	2435.2	2	C
***C*. *yersinii***	5.13 ± 0.04	2.57	2513.5	2	C
***C*. *duplicilobum***	5.22 ± 0.14	2.61	2552.6	2	C
***C*. *birmanicum***	5.28 ± 0.07	2.64	2581.9	2	C
***C*. *arietinum***	5.33	2.67	2611.3	1	C

* number of flow-cytometric measurements (one individual per species was analyzed)

### Molecular phylogeny

The parameters for sequence alignments are given in Table 2. *Cleisostoma yersinii* clustered clearly together with *C*. *birmanicum*, with posterior probability (PP) 1.00 and bootstrap support 100% in maximum parsimony (MP) and maximum likelihood (ML) trees. *Cleisostoma striatum* (Rchb.f.) N.E.Br., another representative of the sect. *Echioglossum* (Bl.) Seidenf., was placed together with *C*. *paniculatum* (Ker Gawl.) Garay (PP 1.00, MP 77%, ML 89%) as a sister group to the *C*. *birmanicum/yersinii* clade (PP 1.00, MP 94%, ML 99%). Evolutionary relationships of this group to other members of the genus are rather unclear. In general, two separate clades were resolved (PP 1.00, MP 77%, ML 97%). The first clade included, in addition to the species of the section *Echioglossum* and several other *Cleisostoma* species, also members of related genera *Pelatantheria* and *Schoenorchis* while the second clade included, in addition to *Cleisostoma*, also species of genera *Cleisocentron*, *Diploprora*, *Malleola*, *Omoea*, *Robiquetia*, *Sarcoglyphis*, *Smitinandia*, *Stereochilus* and *Uncifera*.

**Table 2:** Statistics of alignments used for molecular phylogeny.

	*atpI-atpH*	*nrITS*	*matK*	*psbA-trnH*	*trnL-F*
**number of samples**	42	57	48	41	42
**aligned length**	850	695	1644	846	1352
**no. of variable characters**	284	250	301	123	396
**no. of parsimony-informative characters**	65	135	119	46	172

## Discussion

### Taxonomy and systematics

Only two flowering individuals of *C*. *yersinii* and a few habitually similar sterile plants growing on a roadside together with the type specimen were observed by us. Nevertheless, orchids are known to occur in small and often very sparse populations frequently [44–46]. This is evident also from the fact that many orchid species were described based on the sole specimen or even a single plant in culture which was collected from an unknown place in nature. As an example, we can mention description of a new genus *Thuniopsis* L.Li, D.P.Ye & Shi J.Li which was described in 2015 based on the single herbarium specimen prepared from cultivated plant and only one locality is known in nature to the date [47]. The genus *Kalimantanorchis* Tsukaya was described in 2011 based on the single discovered plant [48]. *Bulbophyllum nocturnum* J.J.Verm., de Vogel, Schuit. & A.Vogel was described based on the three herbarium specimens, nevertheless these were collected from the single cultivated plant collected a few years ago in the wild [49] and many species of this genus are known from the type specimen only [49]. Another example could be a subtribe Pleurothallidinae which has a lot of species described from cultivated material of unknown or inaccurately known wild origin (and perhaps collected only once—e.g. *Dracula deniseana* Luer, *Dracula saulii* Luer & Sijm, *Dracula vinacea* Luer & R. Escobar, *Lepanthes cordeliae* Luer, *Lepanthes persimilis* Luer & Sijm, *Masdevallia princeps* Luer, *Porroglossum tripollex* Luer and many others).

*Cleisostoma yersinii* is most closely related to *C*. *birmanicum*, which occurs from Myanmar through Thailand and Vietnam to Chinese Hainan [2,50]. Both species have the largest flowers in the genus and the lip shape of *C*. *yersinii* is clearly derived from that of *C*. *birmanicum*. Taxonomically-informative characters of both species are summarized in Table 3.

**Table 3:** Comparison of morphological features of *C*. *yersinii* and its putative closest relative *C*. *birmanicum*.

	*Cleisostoma yersinii*	*Cleisostoma birmanicum*
**Leaf shape**	narrowly oblong, 8.5–15.5 × 1.8–2.1 cm	narrowly lanceolate, 13.0–25.0 × 1.0–1.8 cm
**Flower size**	large, up to 2.7 cm wide	smaller, ca. 1.8 cm wide
**Flower opening**	usually 3–4 flowers open at a time	more flowers (often up to ten) open at a time
**Mid-lobe of the lip**	divided into three lobules, margins bent upwards, central lobule S-shaped, projecting into a single short tail	divided into three lobules, central lobe triangular, straight, projecting into two long tails
**Spur**	short and shallow, rather inconspicuous, its function substituted by nectar sacks in the lip	apparent and clearly projecting from the lip, ca. 5 mm long
**Column**	ca. 8 mm long	ca. 4 mm long

Shape of the lip (Figs 3 and 4 and S1 Fig) and leaf shape (Figs 3 and 6) seem to be the most important diagnostic characters. In particular, lip morphology of *C*. *yersinii* is unique among all members of its genus. The function of a spur in directing pollinators is substituted by narrow openings of two nectar sacks that are formed by large lip calli and a septum, and located at the base of a shallow spur. In addition, there is only a single short tail (arista) at the tip of the flower lip of *C*. *yersinii* while two distinctly longer tails are present in *C*. *birmanicum*. The newly described orchid shares with *C*. *birmanicum* the three-lobed lip, but the lip of *C*. *yersinii* is much more complex and bears prominent calli that allow a floral spur to be reduced.

**Fig 8:**
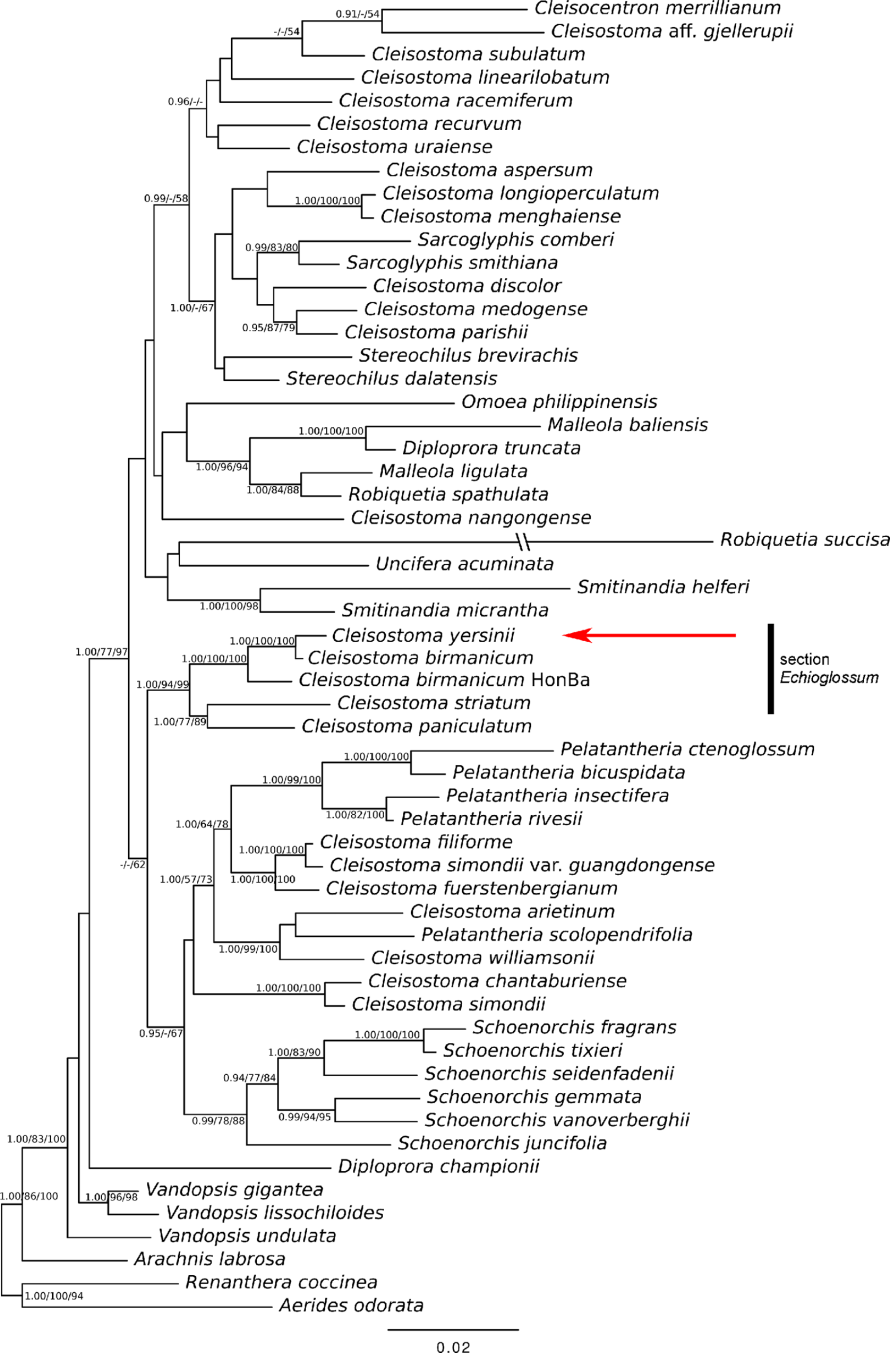
The Bayesian tree obtained from analysis of the combined dataset. The numbers near the nodes are the Bayesian posterior probabilities (at the left) and the bootstrap percentages for Maximum parsimony (in the middle) and Maximum likelihood analysis (at the right). The posterior probabilities and bootstrap supports over 0.9 and 50, respectively, are shown. See S1 Table for GeneBank accession numbers.

Based on morphological similarities with *C*. *birmanicum*, we classify *C*. *yersinii* in sect. *Echioglossum*. Other species belonging to this section are *C*. *javanicum* (Blume) Garay, *C*. *minax* (Rchb.f.) Seidenf. and *C*. *striatum*. The placement of *C*. *yersinii* to sect. *Echioglossum* was clearly supported also by nuclear and plastid sequence data: the newly described species formed a well-supported clade with *C*. *birmanicum* (Fig 8). Surprisingly, one of the two specimens of *C*. *birmanicum* groups closer to *C*. *yersinii* than the other one. This could indicate paraphyly of *C*. *birmanicum*. This species has a relatively wide distribution from Burma through Thailand and Vietnam up to the Chinese island Hainan and occurrence in Cambodia and Laos is expected [2, 50, 51]. However, only further research could shed some light on the classification of *C*. *birmanicum*. The only other species from sect. *Echioglossum* with available molecular data, *C*. *striatum*, formed a well supported group with *C*. *paniculatum*, which belongs to a different section, and this group was sister to the *C*. *birmanicum/yersinii* clade. Similar results were obtained in a recent phylogenetic study based on the combination of data from the same nuclear ITS and plastid regions [9] where *C*. *paniculatum* grouped also together with *C*. *birmanicum* and *C*. *striatum*. While there is little doubt about the placement of *C*. *yersinii* to sect. *Echioglossum*, the monophyly of this section remains to be investigated.

According to [50] members of the sect. *Echioglossum* are characterized by rather stout habit, hippocrepiforme basal part of the stipes of the pollinia, rugose internodes and a double tail at the tip of the lip [50]. All these characters but the double tail are present in *C*. *yersinii*. The tail at the tip of the lip in the newly described species is simple, which requires a slight modification of diagnostic characters of the sect. *Echioglossum*.

It should be noted that the sectional division proposed by [50] is based on morphological similarities and may not reflect evolutionary relationships [cf. 12]. In addition, the author himself considered his infrageneric classification preliminary, based solely on SE Asian, and especially the Thai, samples (i.e., approximately one quarter of the total species diversity). Despite this limitation, the sectional concept seems to be vital for SE Asian species, as demonstrated by taxonomic revisions of Indochinese [51] and Bhutanese [52] taxa.

Below we present updated keys to (i) SE Asian sections (building on [50]) and (ii) Vietnamese species (building on [51]) of the genus *Cleisostoma*. Compared to previously published taxonomic treatments, several newly described species are included while other taxa are reduced to synonymy, and section-specific characters are refined in some cases. In addition to [50,51], the following taxonomic works have been consulted: [2,53–55].

**Table:** 

Identification key to SE Asian sections of the genus *Cleisostoma*.	
1a. Leaves dorsiventral (but occasionally oval or V-shaped in cross-section)	2
1b. Leaves terete	5
2a. Pollinaria with simple (linear or clavate) stipes and undivided viscidium	3
2b. Morphology of stipes and viscidium complex, usually deeply divided into two lobes at the tip with viscidium	4
3a. Leaf apex emarginate	sect. *Cleisostoma*
3b. Leaf apex acute, occasionally with caudate or mucronate tip	sect. *Subulatum* Seidenf.
3b. Leaf apex acute, occasionally with caudate or mucronate tip	sect. *Subulatum* Seidenf.
4a. Lip mid-lobe not extending into the tail	sect. *Paniculatum* Seidenf.
4b. Lip mid-lobe with a single or double tail	sect. *Echioglossum* (Bl.) Seidenf.
5a. Stipes of pollinaria very short (< 0.5 mm), mitre-shaped	sect. *Mitriformes* Seidenf.
5b. Stipes of pollinaria longer and of different shape (linear or more complex)	6
6a. Viscidium discoid, stipes narrowly triangular in front view	sect. *Pilearia* (Lindl.) Seidenf.
6b. Viscidium with two down- and backward-pointing arms, stipes of pollinaria not narrowly triangular in front view	sect. *Complicatum* Seidenf
	(only *C*. *simondii* (Gagnep.) Seidenf. in Vietnam)

**Table:** 

Identification key to Vietnamese species of sect. *Cleisostoma*.	
1a. Lobes of emarginate leaves triangular, acute or acuminate	2
1b. Lobes of emarginate leaves obtuse	3
2a. Mid-lobe of the lip thick, with entire margin, pointing upwards	*C*. *aspersum* (Rchb.f.) Garay
2b. Mid-lobe of the lip thin, with erose front edge, slightly downward-pointing	*C*. *discolor* Lindl.
3a. Leaves 3.0–4.5 cm wide	*C*. *racemiferum* (Lindl.) Garay
3b. Leaves 1.0–2.5 cm wide	4
4a. Tepals greenish to greenish-yellow with a distinctive fine purple markings at the base or in the centre; lip mid-lobe white, tinged violet; side lobes of the lip roundish, orange with two purple lines	*C*. *lendyanum* (Rchb.f.) Garay
4b. Tepals uniformly greenish, greyish-yellow, yellow or pink, occasionally with indistinct purplish median veins or tips but always without contrasting purple markings at the base or in the centre; lip of a different color	5
5a. Stipes of pollinaria exceeding the base of pollinia and viscidium (disc); side lobes of the lip long-triangular, acute, horn-like; flowers light greyish yellow, tip of the mid-lobe purple, tips of side lobes with a single purple dot	*C*. *crochetii* (Guillaumin) Garay
5b. Stipes of pollinaria ending at the base of pollinia and viscidium (disc); side lobes of the lip broadly truncate to truncate; flowers of different color	6
6a. Side lobes of the lip broadly truncate, finely denticulate at the edges; stipes of pollinarium widest in the upper third; flowers dull yellow, side lobes and apex of the lip white	*C*. *flavescens* Aver. & Averyanova
6b. Side lobes of the lip truncate, with entire margins and an inflated callosity at the tip; stipes of pollinarium linear-filiform; flowers light pink occasionally with a yellow tinge, lip purple-violet, side lobes yellowish-pink to orange	*C*. *melanorachis* Aver. & Averyanova

**Table:** 

Identification key to Vietnamese species of sect. *Subulatum*.	
1a. Leaves 4-5 mm wide, succulent, thick and rigid; flowers not widely opening, 2.5–3.0 mm wide	*C*. *subulifolium* Aver. & Averyanova
1b. Leaves wider than 9 mm, thinner; flowers widely opening, 3–9 mm wide	2
2a. Mid-lobe of the lip clearly upward-pointing, with a tip usually bent inwards	*C*. *recurvum* (Hook.) ined.
2b. Mid-lobe of the lip straight (flat) or with only slightly upward-bent margins	3
3a. Leaves narrow (ca. 1.4 cm wide), up to 30 cm long	*C*. *subulatum* Blume
3b. Leaves wider (ca. 2.2 cm wide) and shorter (ca. 10-12 cm long)	*C*. *scortechinii* (Hook.f.) Garay

Note: *C*. *rostratum* (Lindl.) Garay reported from Vietnam by [51] and [24] is considered to be a synonym of *C*. *recurvum* (Hook.) ined. [4]. The latter species was not reported from Vietnam previously.

**Table:** 

Identification key to Vietnamese species of sect. *Paniculatum*.	
1a. Lip mid-lobe flush with the front side of the spur; stipes with very broad upper parts enveloping the pollinia	*C*. *equestre* Seidenf.
1b. Lip mid-lobe oriented at an obtuse angle to spur; stipes not enveloping the pollinia	2
2a. Septum in the spur complete (from wall to wall); inflorescence pendulous	*C*. *duplicilobum* (J.J.Sm.) Garay
2b. Septum in the spur incomplete (occasionally reaching the backwall in distal part of the spur); inflorescence erect to semi-erect	3
3a. Spur distinctly curved forward; mid-lobe of the lip straight, with slightly upward-pointing tip	*C*. *inflatum* (Rolfe) Garay
3b. Spur straight; mid-lobe of the lip distinctly upward-pointing	*C*. *paniculatum* (Ker Gawl.) Garay

Note: *C*. *chapaense* (Guill.) Garay reported from Vietnam by [24] as a separate species is considered to be a synonym of *C*. *paniculatum* (Ker Gawl.) Garay [51].

**Table:** 

Identification key to Vietnamese species of sect. *Echioglossum*.	
1a. Inflorescence shorter than leaves, unbranched; sepals about 6 mm long	*C*. *striatum* (Rchb.f.) N.E.Br.
1b. Inflorescence longer than leaves, branched; sepals 8.5–15 mm long	2
2a. Lateral sepals ca. 9 mm long; tip of the mid-lobe of the lip projecting into two tails; leaves narrowly lanceolate, about 1–1.5 cm wide	*C*. *birmanicum* (Schltr.) Garay
2b. Lateral sepals ca. 14 mm long; tip of the mid-lobe of the lip projecting into a single short tail; leaves narrowly oblong, about 2 cm wide	*C*. *yersinii* J. Ponert & Vuong

**Table:** 

Identification key to Vietnamese species of sect. *Mitriformes*.	
1a. Stem short, erect; leaves thick and short, densely arranged, often recurved	*C*. *arietinum* (Rchb.f.) Garay
1b. Stem long and pendant; leaves thin and long, loosely arranged, usually falcate	*C*. *williamsonii* (Rchb.f.) Garay

**Table:** 

Identification key to Vietnamese species of sect. *Pilearia*.	
1a. Backwall callus pentagonal or heart-shaped with a small vertical median keel; mid-lobe of the lip lilac	*C*. *filiforme* (Lindl.) Garay
1b. Backwall callus three-lobed with side horns; mid-lobe of the lip white	*C*. *fuerstenbergianum* Kraenzl.

### Anatomy

The majority of investigated anatomical characters of *C*. *yersinii* are shared with other orchids belonging to the Vandeae and have no value for phylogenetic reconstructions at intratribal level. Nevertheless, some relatively uncommon anatomical features were found, including the ∩-thickened exodermis cell walls in *C*. *yersinii* roots. While U- and O-thickened cell walls are relatively frequent in the tribe Vandeae, their ∩-thickened counterparts have only been reported in two species out of several dozen investigated: *Dendrophylax lindenii* (Lindl.) Benth. ex Rolfe [20] and *Microcoelia macrantha* (H.Perrier) Summerh. [17]. Both *Dendrophylax* and *Microcoelia* belong to the subtribe Angraecinae [3] while *C*. *yersinii* belongs to the subtribe Aeridinae, indicating that ∩-thickened cell walls in roots may be more widely distributed in the tribe.

Another interesting observation concerns the stomatal density of leaves. The two examined species of sect. *Echioglossum* had stomata on both sides of leaves, while in *C*. *paniculatum* (sect. *Paniculatum*) and *C*. *racemiferum* (sect. *Cleisostoma*) stomata were present on abaxial side only. Hypostomatic species clearly prevail over the amphistomatic ones in the Aeridinae [23,56]. However, both types seem to be scattered along the phylogenetic tree of the Aeridinae tribe [10]. More species need to be investigated to elucidate if stomatal distribution can be used as a marker for delimitation of some groups in the Aeridinae, as appears to be the case of sect. *Echioglossum*.

### Ecology

Several plants of *C*. *yersinii* were found growing as terrestrial on bare mineral soil exposed by road building (Figs 1 and 2). However, their roots spread along the ground surface and did not penetrate into the soil. The ground was only sparsely covered by lichens and mosses, and the growing conditions of *C*. *yersinii* resembled those of epiphytes or lithophytes. In addition, root anatomy showed several adaptations typical of epiphytes, including the two-layered velamen, thickened exodermal cell walls, well-developed pneumatodes and chlorenchymatous cortex [17]. A well-developed leaf hypodermis as observed in *C*. *yersinii* is also unlikely to occur in a terrestrial plant inhabiting wet montane forest. All available evidence thus supports the epiphytic or lithophytic nature of *C*. *yersinii*. Eroded roadsides with bare mineral soil seem to accurately mimic epiphytic conditions and offer a suitable secondary habitat to plants otherwise growing on trees or rocks. Other epiphytic species (e.g., *C*. *birmanicum* and *Thrixspermum annamense*) grew there in sympatry. Primary habitats of the new orchid species are most likely trunks of trees; a single epiphytic plant was recorded close to *locus classicus* in 2014.

### Conservation status

The species is known only from the type locality and a single plant observed two years later close to the original site. This could indicate its rarity although new localities will likely be found in the little explored and hardly accessible Hon Ba mountains. At this stage of investigation, we regard *C*. *yersinii* as “Data Deficient” according to the IUCN Red List categories [57].

The Hon Ba Mts. is isolated from most other mountain ranges by quite extensive lowlands. The only mountains connected by more or less continuous mountain ridge are the Da Lat and the Hon Giao areas, both of which have been studied relatively intensively in the past, with no records of the newly described species. This may indicate that *C*. *yersinii* is a narrow endemic of the Hon Ba Nature Reserve. In such a case, the species would qualify as “Threatened” because Hon Ba forests are under high pressure through illegal logging.

### Nectar characteristics and pollination

The lack of fruits from flowers not subjected to hand-pollination under the greenhouse conditions suggests allogamy. This assumption is supported by relatively large and colorful flowers with complicated morphology that would unlikely evolved without interactions with pollinators [58].

Sugar-rich nectar with dominant sucrose and lower amounts of fructose and glucose, as detected in *C*. *yersinii*, is common in Orchidaceae, although raffinose and some other minority sugars have also been found in other orchid species [59]. However, we cannot exclude the possibility that additional sugars were present in the nectar of *C*. *yersinii* in concentrations under our detection limit.

Nectar composition is known to be affected by pollinator feeding [60,61], either by feeding itself or by pollinator-borne microorganisms [60,62–64]. On the other hand, nectar composition seems to be relatively stable under controlled conditions where interacting (micro)organisms are avoided. We argue that our measurements were not influenced by pollinators as the greenhouse was pollinator-free and the sugar composition varied only little between the samples. Nectarivorous microorganisms are able to induce hydrolysis of sucrose into hexoses, from which they preferably utilize glucose. This way seems to be the only route for a fructose-dominant nectar in plants [65,66]. Slightly higher concentration of glucose over fructose in our samples suggests no effect of nectarivorous microorganisms on sugar composition. Low amounts of hexoses found in the nectar of *C*. *yersinii* most likely originated by spontaneous or plant-induced hydrolysis of sucrose, as previously reported in other plants [66]. In some African hawkmoth-pollinated orchids with long spurs, a gradient from a low sugar concentration at the mouth of the spur to a high sugar concentration at the tip was observed, functioning as a ‘sugar trail’ enticing long-tongued hawkmoths to probe deeply into spurs [67]. It is, however, unlikely that such gradient could exist in flowers of *C*. *yersinii* that have relatively wide, suborbicular nectar sacs.

As indicated above, variation in nectar sugar composition may be very high under natural conditions [60,68–70], occasionally even higher among different flowers of the same individual than between individuals or populations [65]. Under such conditions, little relationship between nectar composition and pollination syndrome is expected (e.g., [71,72]). However, in other cases, nectar characteristics may be useful in inferring putative pollinators. Considering the flower size, lip shape and color, birds and bats should be excluded as potential pollinators of *C*. *yersinii*. Butterflies are also unlikely to pollinate the flowers because of a massive and wide viscidium. The most likely pollinators of the newly recognized species seem to be bees. This group of insects prefers nectar with dominant sucrose whereas flies and several Hymenoptera prefer hexoses-dominant nectar [see 72 and references therein]. As compared to butterflies and moths, bees (e.g., *Apis* and *Bombus*) are known to favor more concentrated nectar [73], providing further evidence for their role in the pollination biology of *C*. *yersinii*. Field observations are, however, necessary to conclusively determine the identity of pollinators.

## Supporting Information

S1 Fig Close-up of the lip of *Cleisostoma yersinii*. Scale bars 1 mm. A specimen cultivated in the Prague Botanical Garden collected as holotype. Photo J. Ponert.
(TIF)

S2 Fig *Cleisostoma yersinii* growing as an epiphyte. Primary submontane evergreen forest at elevation about 1500 m a.s.l. Photo T.B.Wuong.
(TIF)

S3 Fig Chromatogram from HPLC analysis of saccharides in the nectar of *Cleisostoma yersinii*. The first dominant peak corresponds to sucrose while the second and the third minority peaks correspond to glucose and fructose, respectively.
(TIF)

S1 Table Plant samples used in molecular phylogenetic analysis. Accession numbers in the living collection of the Prague Botanical Garden and original localities are provided for the newly sequenced *Cleistostoma* species.
(DOC)
