# Supplementary figures and images for: A New Species of Cleisostoma (Orchidaceae) from the Hon Ba Nature Reserve in Vietnam: A Multidisciplinary Assessment
Source: PLoS One. 2016 Mar 23;11(3):e0150631. doi: 10.1371/journal.pone.0150631 (PMC4805174; doi:10.1371/journal.pone.0150631)

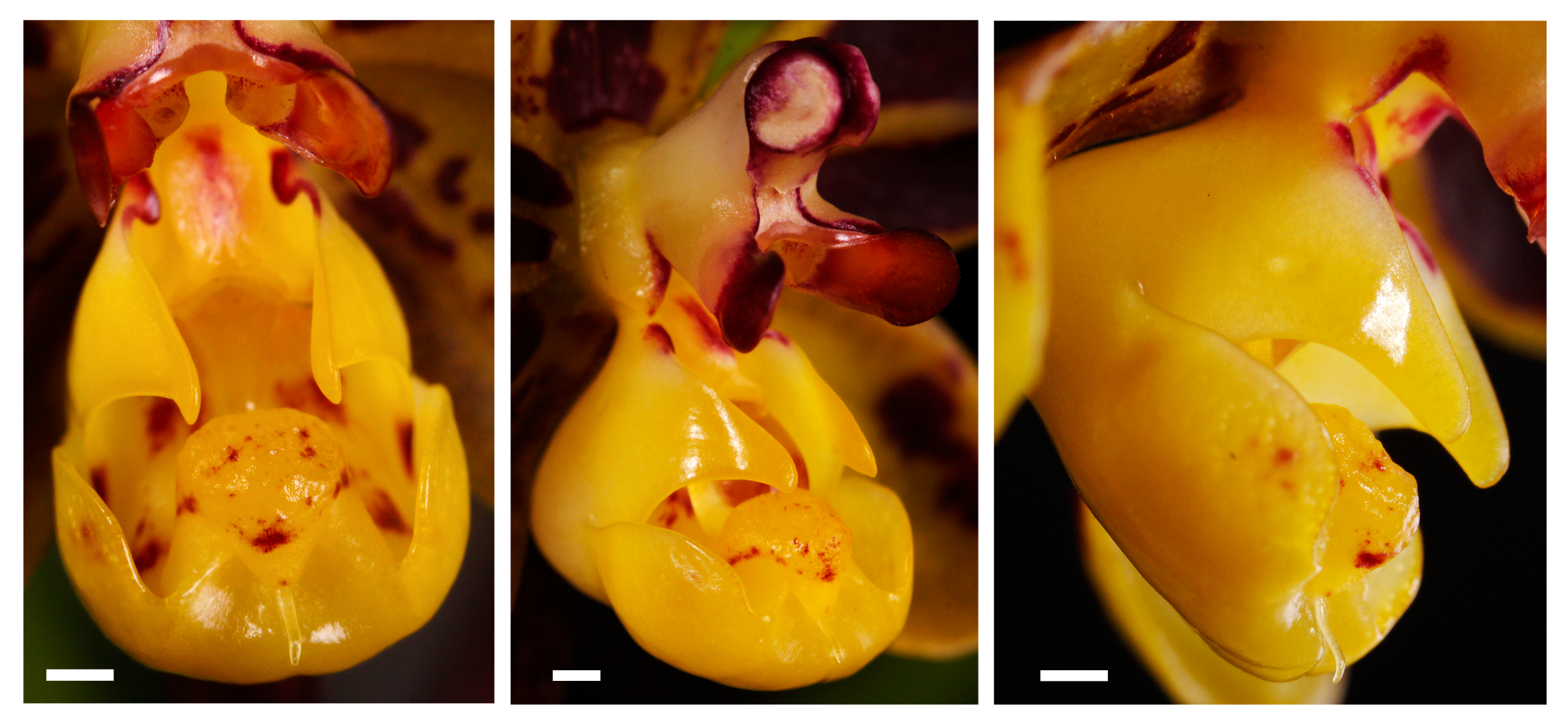

Supplement: S1 Fig — Scale bars 1 mm. A specimen cultivated in the Prague Botanical Garden collected as holotype. Photo J. Ponert. (TIF) [file pone.0150631.s001.tif]

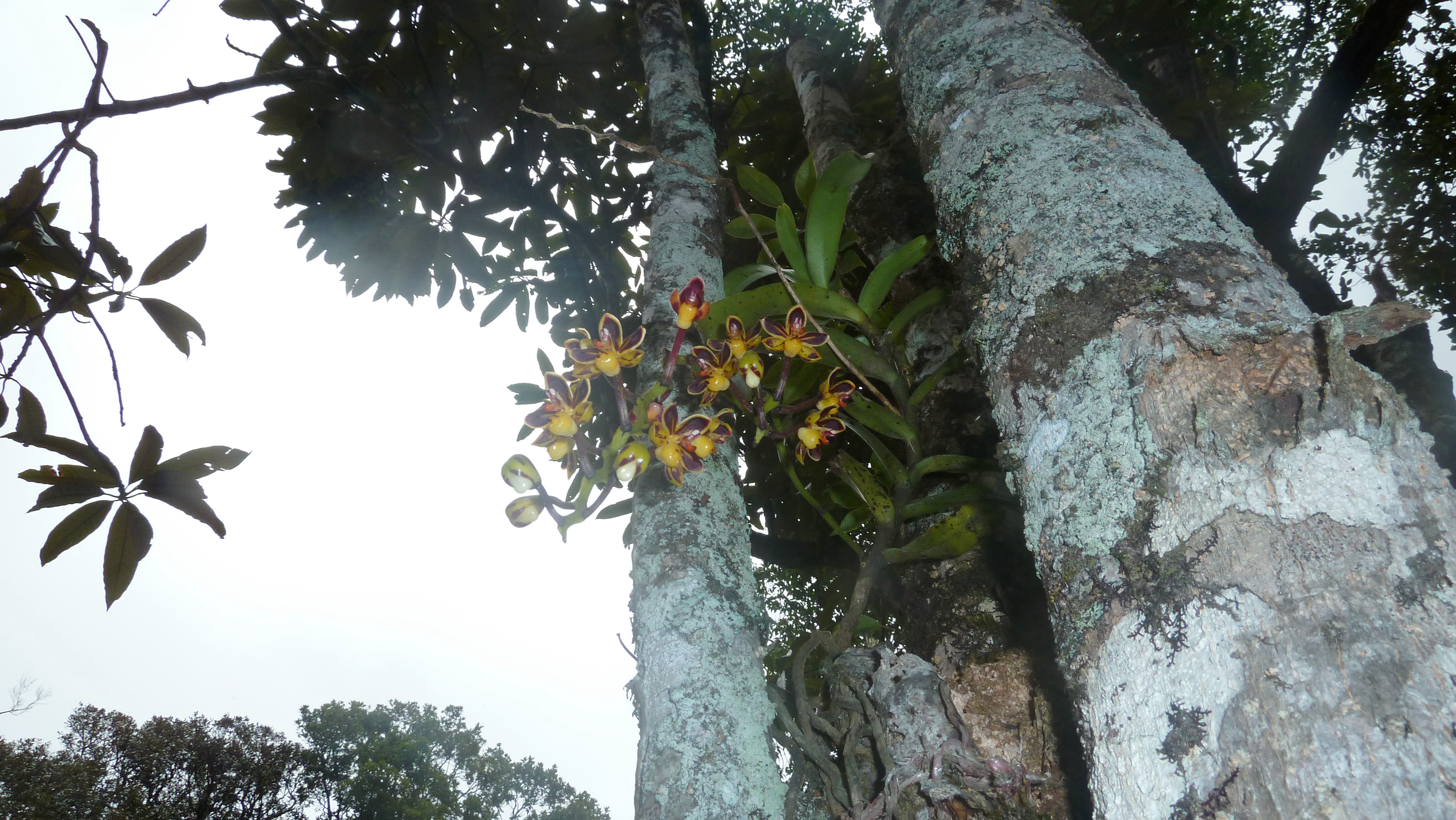

Supplement: S2 Fig — Primary submontane evergreen forest at elevation about 1500 m a.s.l. Photo T.B.Wuong. (TIF) [file pone.0150631.s002.tif]

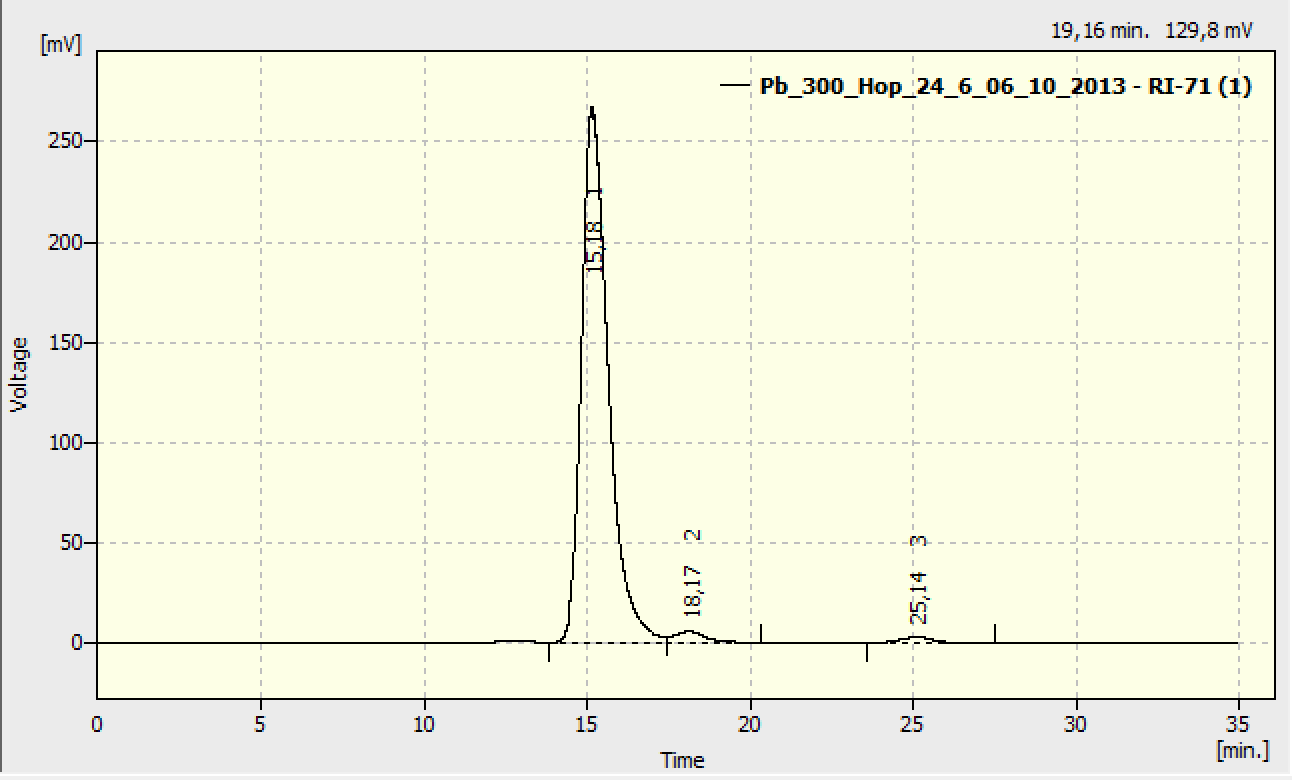

Supplement: S3 Fig — The first dominant peak corresponds to sucrose while the second and the third minority peaks correspond to glucose and fructose, respectively. (TIF) [file pone.0150631.s003.tif]
