# Supplementary material for: A New Species of Cleisostoma (Orchidaceae) from the Hon Ba Nature Reserve in Vietnam: A Multidisciplinary Assessment
Source: PLoS One. 2016 Mar 23;11(3):e0150631. doi: 10.1371/journal.pone.0150631 (PMC4805174; doi:10.1371/journal.pone.0150631)
Supplement: S1 Table — Accession numbers in the living collection of the Prague Botanical Garden and original localities are provided for the newly sequenced Cleistostoma species. (DOC) [file pone.0150631.s004.doc]

Supplementary table S1.

| **Species** | **Herbarium voucher** | **locality detail (Prague Botanical Garden accession number)** | **nrITS** | ***atpI*-*atpH*** | ***matK*** | ***psbA*-*trnH*** | ***trnL*-*F*** |
| --- | --- | --- | --- | --- | --- | --- | --- |
| *Aerides odorata* Lour. | Z. J. Liu5126 (NOCC) |  | KC244654 | KC244670 | KC244658 | KC244666 | KC244662 |
| *Arachnis labrosa* (Lindl. & Paxton) Rchb.f. | Z. J. Liu 200177 (NOCC) |  | KJ733386 | KJ733308 | KJ733545 | KJ733466 | KJ733623 |
| *Cleisocentron merrillianum* (Ames) Christenson | TBG137038 (TNS) |  | AB217541 | - | - | - | - |
| *Cleisostoma aff.gjellerupii* (J.J.Sm.) Garay |  |  | AB217545 | - | AB217721 | - | - |
| *Cleisostoma arietinum* (Rchb.f.) Garay | Z. J. Liu 6991 (NOCC) |  | KJ733391 | KJ733313 | KJ733550 | KJ733471 | KJ733628 |
| *Cleisostoma aspersum* (Rchb.f.) Garay | Z. J. Liu 4483 (NOCC) |  | KJ733392 | KJ733314 | KJ733551 | KJ733472 | KJ733629 |
| *Cleisostoma birmanicum* (Schltr.) Garay | Z. J. Liu 4599 (NOCC) |  | KJ733393 | KJ733315 | KJ733552 | KJ733473 | KJ733630 |
| *Cleisostoma birmanicum* (Schltr.) Garay |  | Vietnam, Khanh Hoa, Hon Ba (2012.10822) | KT223751 | KU342565 | KU342567 | KU342569 | KU342571 |
| *Cleisostoma chantaburiense* Seidenf. | Carlsward 155 (SEL) |  | DQ091695 | - | - | - | - |
| *Cleisostoma discolor* Lindl. | Carlsward 253 (FLAS) |  | DQ091696 | - | - | - | - |
| *Cleisostoma filiforme* (Lindl.) Garay | Z. J. Liu 4579 (NOCC) |  | KJ733394 | KJ733316 | KJ733553 | KJ733474 | KJ733631 |
| *Cleisostoma fuerstenbergianum* Kraenzl. | Z. J. Liu 3891 (NOCC) |  | KJ733395 | KJ733317 | KJ733554 | KJ733475 | KJ733632 |
| *Cleisostoma linearilobatum* (Seidenf. & Smitinand) Garay | Z. J. Liu 7117 (NOCC) |  | KJ733396 | KJ733318 | KJ733555 | KJ733476 | KJ733633 |
| *Cleisostoma longioperculatum* Z.H.Tsi | Z. J. Liu 2717 (NOCC) |  | KJ733397 | KJ733319 | KJ733556 | KJ733477 | KJ733634 |
| *Cleisostoma medogense* Z.H.Tsi | Z. J. Liu 4188 (NOCC) |  | KJ733398 | KJ733320 | KJ733619 | KJ733478 | KJ733635 |
| *Cleisostoma menghaiense* Z.H.Tsi | Z. J. Liu 4173 (NOCC) |  | KJ733399 | KJ733321 | KJ733557 | KJ733479 | KJ733636 |
| *Cleisostoma nangongense* Z.H.Tsi | Z. J. Liu 3600 (NOCC) |  | KJ733400 | KJ733322 | KJ733558 | KJ733480 | KJ733637 |
| *Cleisostoma paniculatum* (Ker Gawl.) Garay | Z. J. Liu 2400 (NOCC) |  | KJ733401 | KJ733323 | KJ733559 | KJ733481 | KJ733638 |
| *Cleisostoma parishii* (Hook.f.) Garay | Z. J. Liu 4597 (NOCC) |  | KJ733402 | KJ733324 | KJ733560 | KJ733482 | KJ733639 |
| *Cleisostoma racemiferum* (Lindl.) Garay | Z. J. Liu 6507 (NOCC) |  | KJ733403 | KJ733325 | KJ733561 | KJ733483 | KJ733640 |
| *Cleisostoma recurvum* (Hook.) ined. | Z. J. Liu 2508 (NOCC) |  | KJ733404 | KJ733326 | KJ733562 | KJ733484 | KJ733641 |
| *Cleisostoma simondii* (Gagnep.) Seidenf. | Z. J. Liu 7441 (NOCC) |  | KJ733405 | KJ733327 | KJ733563 | KJ733485 | KJ733642 |
| *Cleisostoma simondii var. guangdongense* Z.H.Tsi | Z. J. Liu 2592 (NOCC) |  | KJ733406 | KJ733328 | KJ733564 | KJ733486 | KJ733643 |
| *Cleisostoma striatum* (Rchb.f.) N.E.Br. | Z. J. Liu 3983 (NOCC) |  | KJ733407 | KJ733329 | KJ733565 | KJ733487 | KJ733644 |
| *Cleisostoma subulatum* Blume |  | Vietnam, Dong Nai prov., 75 km NE of Saigon, Tri An (2000.19141) | KT223753 | - | - | - | - |
| *Cleisostoma uraiense* (Hayata) Garay & H.R.Sweet | OM03925 |  | KJ733408 | KJ733330 | KJ733566 | KJ733488 | KJ733645 |
| *Cleisostoma williamsonii* (Rchb.f.) Garay | Z. J. Liu 4525 (NOCC) |  | KJ733409 | KJ733331 | KJ733567 | KJ733489 | KJ733646 |
| *Cleisostoma yersinii* J.Ponert | Jan Ponert 674 (PRC) | Vietnam, Khanh Hoa, Hon Ba (2012.10823) | KT223754 | KU342566 | KU342568 | KU342570 | KU342572 |
| *Diploprora championii* (Lindl.) Hook.f. | Z. J. Liu 4480 (NOCC) |  | KJ733410 | KJ733332 | KJ733568 | KJ733490 | KJ733647 |
| *Diploprora truncata* Rolfe ex Downie | TBG133822 (TNS) |  | AB217549 | - | AB217725 | - | - |
| *Malleola baliensis* J.J.Sm. | TBG127481 (TNS) |  | AB217561 | - | AB217737 | - | - |
| *Malleola ligulata* (J.J.Sm.) J.J.Sm. | Carlsward 246 (FLAS) |  | DQ091700 | - |  | - | - |
| *Omoea philippinensis* Ames | TBG133261 (TNS) |  | AB217566 | - | - | - | - |
| *Pelatantheria bicuspidata* Tang & F.T.Wang | Z. J. Liu 7114 (NOCC) |  | KJ733428 | KJ733349 | KJ733585 | KJ733508 | KJ733665 |
| *Pelatantheria ctenoglossum* Ridl. | TBG130214 (TNS) |  | AB217569 | - | - | - | - |
| *Pelatantheria insectifera* (Rchb.f.) Ridl. | Carlsward 164 (SEL) |  | DQ091692 | - | - | - | GU185931 |
| *Pelatantheria rivesii* (Guillaumin) Tang & F.T.Wang | Z. J. Liu 2518 (NOCC) |  | KJ733429 | KJ733350 | KJ733586 | KJ733509 | KJ733666 |
| *Pelatantheria scolopendrifolia* (Makino) Aver. | Z. J. Liu 6058 (NOCC) |  | KJ733430 | KJ733351 | KJ733587 | KJ733510 | KJ733667 |
| *Renanthera coccinea* Lour. | Z. J. Liu 21117 (NOCC) |  | KJ733441 | KJ733362 | KJ733598 | KJ733521 | KJ733678 |
| *Robiquetia spathulata* (Blume) J.J.Sm. | Z. J. Liu 6691 (NOCC) |  | KJ733443 | KJ733364 | KJ733600 | KJ733523 | KJ733680 |
| *Robiquetia succisa* (Lindl.) Seidenf. & Garay | Z. J. Liu 5248 (NOCC) |  | KJ733444 | KJ733365 | KJ733601 | KJ733524 | KJ733681 |
| *Sarcoglyphis comberi* (J.J.Wood) J.J.Wood | Carlsward 296 (FLAS) |  | DQ091697 | - | AB217759 | - | - |
| *Sarcoglyphis smithiana* (Kerr) Seidenf. | Z. J. Liu 4192 (NOCC) |  | KJ733445 | KJ733366 | KJ733602 | KJ733525 | KJ733682 |
| *Schoenorchis fragrans* (C.S.P.Parish & Rchb.f.) Seidenf. & Smitinand | Z. J. Liu 3881 (NOCC) |  | KJ733446 | KJ733367 | KJ733603 | KJ733526 | KJ733683 |
| *Schoenorchis gemmata* (Lindl.) J.J.Sm. | Z. J. Liu 3977 (NOCC) |  | KJ733447 | KJ733368 | KJ733604 | KJ733527 | KJ733684 |
| *Schoenorchis juncifolia* Reinw. ex Blume | Whitten 1783 (FLAS) |  | DQ091704 | - | - | - | - |
| *Schoenorchis seidenfadenii* Pradhan | Z. J. Liu 5230 (NOCC) |  | KJ733448 | KJ733369 | KJ733605 | KJ733528 | KJ733685 |
| *Schoenorchis tixieri* (Guillaumin) Seidenf. | Z. J. Liu 4123 (NOCC) |  | KJ733449 | KJ733370 | KJ733606 | KJ733529 | KJ733686 |
| *Schoenorchis vanoverberghii* Ames | OM09878 |  | KJ733450 | KJ733371 | KJ733607 | KJ733530 | KJ733687 |
| *Smitinandia helferi* (Hook.f.) Garay | TBG140484 (TNS) |  | AB217587 | - | AB217763 | - |  |
| *Smitinandia micrantha* (Lindl.) Holttum | Z. J. Liu 21119 (NOCC) |  | KJ733451 | KJ733372 | KJ733608 | KJ733531 | - |
| *Stereochilus brevirachis* Christenson | Z. J. Liu 4485 (NOCC) |  | KJ733453 | KJ733374 | KJ733610 | KJ733533 | KJ733689 |
| *Stereochilus dalatensis* (Guillaumin) Garay | Z. J. Liu 5392 (NOCC) |  | KJ733454 | KJ733375 | KJ733611 | KJ733534 | - |
| *Uncifera acuminata* Lindl. | Z. J. Liu 6239 (NOCC) |  | KJ733461 | KJ733380 | KJ733616 | - | KJ733696 |
| *Vandopsis gigantea* (Lindl.) Pfitzer | Z. J. Liu 3275 (NOCC) |  | KJ733462 | KJ733381 | KJ733617 | KJ733541 | KJ733697 |
| *Vandopsis lissochiloides* (Gaudich.) Pfitzer |  |  | EF670377 | - | EF655806 | - | EF670418 |
| *Vandopsis undulata* (Lindl.) J.J.Sm. | Z. J. Liu 3727 (NOCC) |  | KJ733463 | KJ733382 | KJ733618 | KJ733542 | KJ733698 |

**S1 Table. Plant samples used in molecular phylogenetic analysis.** GenBank accession numbers are given for all sequences. Accession numbers in the living collection of the Prague Botanical Garden and original localities are provided for the newly sequenced *Cleistostoma* species.
